# Lossless Compression for the Model Context Protocol: Energy Consumption, Latency, and Bandwidth Trade-Offs

**DOI:** 10.3390/s26144582

**Published:** 2026-07-20

**Authors:** Kalyani Rohidas Vaidya, Dhanalakshmi Kannur Munirathnam, Patrick Seeling

**Affiliations:** Department of Computer Science, Central Michigan University, Mount Pleasant, MI 48859, USA; vaidy1k@cmich.edu (K.R.V.); kannu1d@cmich.edu (D.K.M.)

**Keywords:** Model Context Protocol (MCP), data compression, zstandard, brotli, lz4, gzip, bandwidth optimization, edge devices, large language model agents

## Abstract

As Large Language Models (LLMs) increasingly operate in agentic environments communicating via the Model Context Protocol (MCP), the structured JSON-RPC message traffic is growing rapidly. Yet the current MCP specification does not include any compression mechanism, leaving potential bandwidth optimizations unaddressed. This paper presents the first empirical energy measurement study of lossless compression applied to MCP payloads on embedded ARM hardware. Four production codecs are evaluated at a single, consistent real-time operating point per codec (LZ4 L0, Zstd L3, gzip L6, Brotli Q4). Across two different datasets, our results show that compression is counterproductive below approximately 50–200 bytes depending on the codec, while savings of up to 50% are achievable for larger payloads. Brotli achieves the highest compression ratio (49.9% combined savings) but incurs the greatest CPU and energy cost. Zstd offers the best practical trade-off, delivering approximately 47% bandwidth reduction with low overhead. Message size is the dominant predictor: below a per-codec break-even of approx. 200 B for LZ4, compression expands rather than shrinks the payload. The apparent per-message-type penalties reflect the prevalence of short messages, not the codec. Message size is identified as the primary predictor of compression benefit, and codec selection guidelines are provided for latency-critical, general-purpose, and bandwidth-constrained MCP deployments on resource-constrained devices.

## 1. Introduction

Large Language Models (LLMs) are rapidly evolving from passive text generators into active agents that interact with external systems through structured tool calls [1,2]. Anthropic’s Model Context Protocol (MCP), released in late 2024 [3], defines a schema-driven standard for dynamic tool discovery and invocation that is quickly gaining industry adoption [4,5]. Initially, creating MCP servers remained a largely manual process: developers were required to implement boilerplate integration code, map Application Programming Interface (API) schemas to MCP tool definitions, and handle authentication and serialization by hand. This approach effectively replicates integration efforts that MCP was designed to eliminate [5] and hinders broad adoption.

Newer approaches provide automation support to alleviate the cumbersome approach of API–MCP integration, which will greatly simplify new implementations at the network edge and in sensor-based systems: (i) ToolLLM [1] shows that open-source LLMs can reliably call real-world APIs; (ii) RestGPT [2] extends this to real-world RESTful APIs using a coarse-to-fine online planner whose API Selector uses OpenAPI Specifications (OAS); (iii) OASBuilder [6] converts unstructured HTML API docs into machine-readable OAS via LLM-based extraction and Selenium [7] scraping; (iv) the ACE framework [8] further enriches tool definitions with natural-language descriptions; and (v) ToolFactory [9] consumes OAS-like JSON schemas to produce executable LangChain-compatible tools. The MCP landscape survey [4] characterizes the full lifecycle of an MCP server (creation, deployment, operation, and maintenance) and identifies 16 security threat scenarios across four attacker classes. Overall, these efforts indicate that MCP-supported invocation of APIs and offering of MCP-supported services will increase. In turn, this will also increase the related network traffic to exchange messages and invoke toolchains.

MCP messages are encoded in JSON-RPC 2.0 and transmitted over stdio or HTTP-SSE transports. As tool payloads grow, for example, with resource listings, structured API responses, bulk data, and inter-tool communications, uncompressed JSON becomes a bottleneck, especially on bandwidth-constrained or high-latency connections. Compression on the application layer using HTTP is commonplace, and would also cover JSON-encoded MCP messages. Beyond general-purpose codecs, binary serializations of the JSON data model, e.g., CBOR [10], target structured-protocol payloads directly, while existing approaches to lossless compression for wireless-sensor and IoT nodes [11,12], including its energy cost on constrained devices [13], establish compression as an energy-management tool at the edge. The MCP specification does not, at the time of writing, contain any compression features. Our work addresses this current gap in the characterization of compression trade-offs, specifically within the MCP message corpus.

## 2. Materials and Methods

In this section, we highlight experimental validation of our prior discussions.

### 2.1. MCP Source Data

We combine a generic evaluation of the compression with a practical real-world tool-call approach. We combined the MCP trace dataset [14] with a lightweight OpenInference-style capture of representative MCP tool-call requests, tool results, and error responses [15]. The final combined dataset contains 702 messages, and 100 measured runs were performed per configuration, including warm-up runs to reduce timing noise.

We furthermore employed the Scale.AI MCP Atlas Benchmark [16] dataset’s 500 sample task queries and final responses. The dataset is publicly available from https://huggingface.co/datasets/ScaleAI/MCP-Atlas (accessed on 5 July 2026) for reproducibility in performance measurements. The MCP sample comprised 500 MCP tool-call request payloads (queries, mean 896 bytes, range 511–2680 bytes) and 500 tool-result final result response payloads (responses, mean 468 bytes, range 239–3100 bytes). We pre-processed these by adding an identifier and the message provided by the dataset and encoded them into JSON format. Response payloads were pre-compressed offline using one of the compression algorithms with the same settings used for queries. This way, the compression overhead does not affect measurements.

#### Codecs Under Experimental Test

Five configurations were evaluated: an uncompressed baseline (Raw) and four production-grade lossless codecs applicable to real-time protocol compression. All four codecs are standardized or de facto standards with production-ready Python 3.14 bindings. We provide an overview in Table 1 for the selected codecs that all should support real-time scenarios.

Raw transmission with no content encoding represents the current default MCP deployment: the MCP specification (v2024-11-05) does not mandate or recommend any compression scheme. LZ4 is a byte-oriented LZ77 derivative designed to saturate memory bandwidth rather than maximize compression ratio [17]. At Level 0 (the fastmode), it achieves compression throughput exceeding 500 MB/s and decompression exceeding 1 GB/s on modern Cortex-A-class processors [18]. LZ4 (4.4.5/liblz4 1.9.4 and 1.10.0 as native C module) thus represents the minimum-overhead end of the practical compression space. For MCP deployments where sub-millisecond per-message latency budgets must be preserved, any codec should impose Central Processing Unit (CPU) overhead smaller than the round-trip time (RTT) itself. Zstandard (Zstd, 0.25.0/libzstd 1.5.7) was developed at Meta (formerly Facebook) as a successor to zlib, targeting a better speed–ratio trade-off than either LZ4 or DEFLATE [19]. It was standardized by the Internet Engineering Task Force (IETF) in 2021 as Request for Comment (RFC) 8878 [20]. Level 3 is the RFC-designated default and the level used by the Linux kernel’s Zstd integration. As Zstd is increasingly adopted for application-layer protocol compression, it represents a common general-purpose choice. gzip (stdlib and stock DEFLATE) is built on the DEFLATE algorithm [21] and is specified in RFC 1952 [22]. It is the de facto standard for HyperText Transfer Protocol (HTTP) content encoding, and the Content-Encoding: gzip header is supported by every HTTP/1.1 client and server since RFC 2616 [23]. It is available in the Python standard library without any third-party dependencies, and the commonly observed level is 6. gzip represents a second common alternative for potential deployment, as any HTTP implementation typically already supports it. Brotli (1.2.0) was developed at Google and standardized as RFC 7932 [24]. Brotli combines LZ77 back-references with Huffman coding and additional features [25] and is very efficient but requires higher compute levels. Brotli is natively supported in all major browsers and web servers and defines quality levels from Q0–Q11. The quality level Q4 we adopted is the upper bound of Brotli’s streaming quality range (Q0–Q4), suited for a bandwidth-constrained deployment scenario.

### 2.2. Experimental Setup

**Figure 1:**
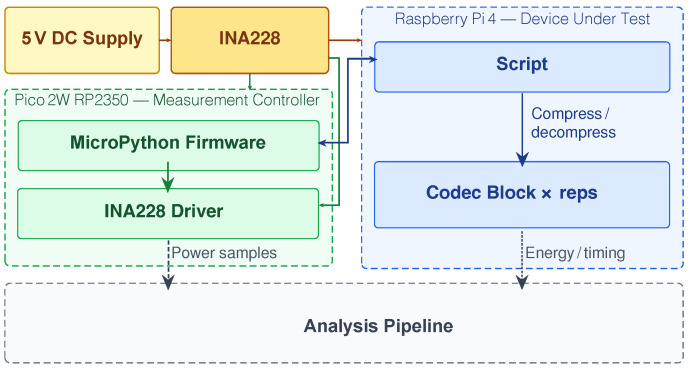
Hardware measurement setup for the MCP payload compression benchmark using the Raspberry Pi 4 as DUT. The INA228 power monitor is wired in-series on the 5 V supply powering the DUT. The Raspberry Pi Pico 2W (RP2350), located left of the DUT, reads the INA228 and sends the samples to the desktop for analysis while receiving GPIO pulses from the DUT as measurement markers.

Compression measurements were performed either on an Apple MacBook Pro M4 (Apple Inc., Cupertino, CA, USA) or on a Raspberry Pi 4 Model B (Raspberry Pi Ltd., Cambridge, UK, with BCM2711, quad-core Cortex-A72, 1.5 GHz, 4 GB LPDDR4) as the device under test (DUT) running DietPi (Linux kernel 6.12) with CPU frequency locked to 1.5 GHz via cpufreq-set. Thus, performance metrics, such as compression time and energy measurements, are more tightly controlled. We additionally perform limited measurements employing a Raspberry Pi Pico 2W (Raspberry Pi Ltd., Cambridge, UK, with RP2350, dual Cortex-M33 at 150 MHz, 520 kB SRAM) running MicroPython v1.28.0 as DUT. We illustrate the overall performance measurement setup in Figure 1 for the Raspberry Pi 4 (RPi4) setup.

The DUTs were powered via an INA228 breakout board (Adafruit Industries, New York, NY, USA) that sampled at over 1 kHz using an independently powered Raspberry Pi Pico 2W (RP2350) as a dedicated measurement controller. We validated the INA228 against an external multimeter wired in series with the 5 V supply; at idle the two agreed to within measurement tolerance (just under 7%). A desktop computer acted as the orchestrator and precompressed response server. In addition to on-device compression, the RPi4 enabled its WLAN interface and transmitted the (compressed) MCP payloads to the desktop. The wireless interface was brought down before the compression phase and only activated for the transmission part of the measurement study to eliminate 802.11 association noise from the Central Processing Unit (CPU) energy signal. An idle baseline was measured in a 5 s window with WiFi disabled before the first benchmark run. Net energy per window subtracts the idle power integrated over the window, $E_{\mathrm{net}}=E_{\mathrm{gross}}-P_{\mathrm{idle}}\cdot t_{\mathrm{window}}$. We note that we locked the CPU governor to 1.5 GHz for reproducibility, which resulted in about 2.2 W idle power, consistent with a typical Raspberry Pi 4. Orchestration relied on a direct Ethernet or UART/FTDI link to the desktop. Each compression-ratio configuration was preceded by 10 unrecorded warm-up runs (priming caches and verifying round-trip correctness) before 100 measured runs; the Raspberry Pi energy measurements recorded all repeats under the locked CPU governor with idle-power subtraction; and on the Pico a cold-start batch was discarded, retaining four warm batches. The development of measurement scripts was assisted by Anthropic Claude Opus 4.6.

## 3. Results

### 3.1. Generic Compression Results

**Figure 2:**
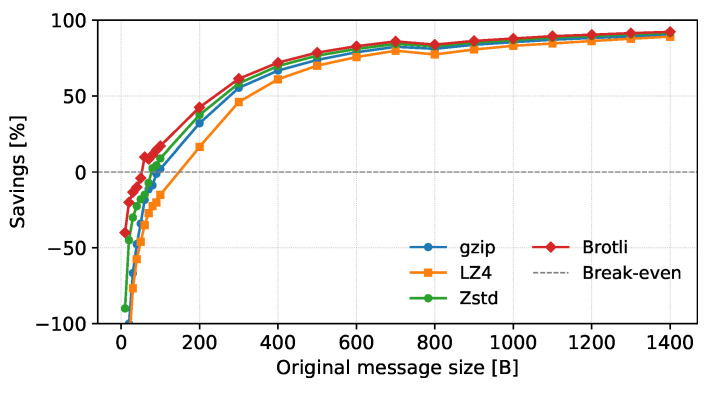
Generic content compression savings by algorithm.

For a first evaluation of attainable compression, we initially generate a synthetic MCP message corpus ranging from 10 to 1400 bytes, representative of tool call messages, resource listings, and structured API responses encountered in practice. Each message was compressed, and the compression ratio was recorded. The break-even message size for each algorithm can be defined as the smallest message size at which space savings are positive. All benchmarks were performed using Python 3.14/MicroPython v1.28.0 with gzip (stdlib on the RPi4 and stock DEFLATE on the Pico), zstandard (0.25.0/libzstd 1.5.7), lz4 (4.4.5/liblz4 1.9.4 on the RPi4 and 1.10.0 native C module on the Pico), and brotli (1.2.0) PyPI packages at the matched real-time operating points (LZ4 L0, Zstd L3, gzip L6, Brotli Q4). We illustrate this initial evaluation in Figure 2.

We initially observe that for messages below about 50–100 B, all algorithms increase message size due to header overhead. For example, at 10 B, gzip produces 30 B (−200%), LZ4 produces 33 B (−230%), Zstd 19 B (−90%), and Brotli 14 B (−40%). MCP tool-call acknowledgments and short ping messages should therefore not be compressed. The break-even message sizes, i.e., where savings ≥0%, that we observed were Brotli ≈ 60 B, Zstd ≈ 80 B, gzip ≈ 100 B, and LZ4 ≈ 200 B. Brotli’s lower break-even reflects its richer built-in static dictionary. At 1400 B the observed ordering by space savings is Brotli (92.3%) > Zstd (91.71%) > gzip (90.43%) > LZ4 (89.14%). The gap narrows at large sizes as all algorithms exploit JSON’s high structural redundancy.

### 3.2. MCP Trace Dataset

**Figure 3:**
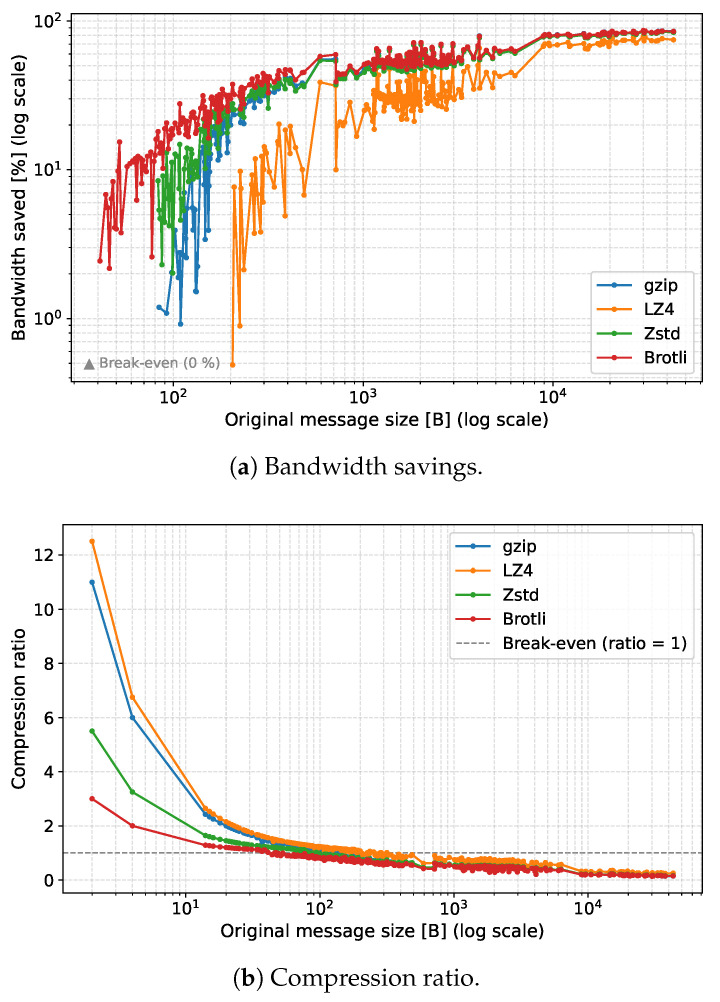
Bandwidth savings and compression ratio for the trace dataset.

For this specific evaluation, we employ the trace dataset on a MacBook with the same matched real-time operating points used throughout (LZ4 L0, Zstd L3, gzip L6, Brotli Q4). We initially observe the attained savings and compression ratio in Figure 3a and Figure 3b, respectively.

We initially notice a dominant trend of compression savings increasing monotonically with message size. For messages below about 20 B, all codecs produce negative savings as the compressed output is larger than the input due to framing headers. Brotli crosses break-even earliest (around 30–40 B), Zstd at about 70 B, gzip at about 100 B, and LZ4 not until about 200 B. Above 1 kB, gzip, Zstd, and Brotli cluster tightly and are nearly indistinguishable. LZ4 commonly performs about 10–20% below the other evaluated codecs. The resulting compression ratio (compressed/original size) illustrates the clear delineation between compression overhead and useful compression. At 3–5 B message sizes, gzip and LZ4 produce output that is an order of magnitude larger. Depending on the codec, they yield positive compression results (ratio below one) around 50–200 B. Brotli consistently achieves the lowest ratio at every size.

**Figure 4:**
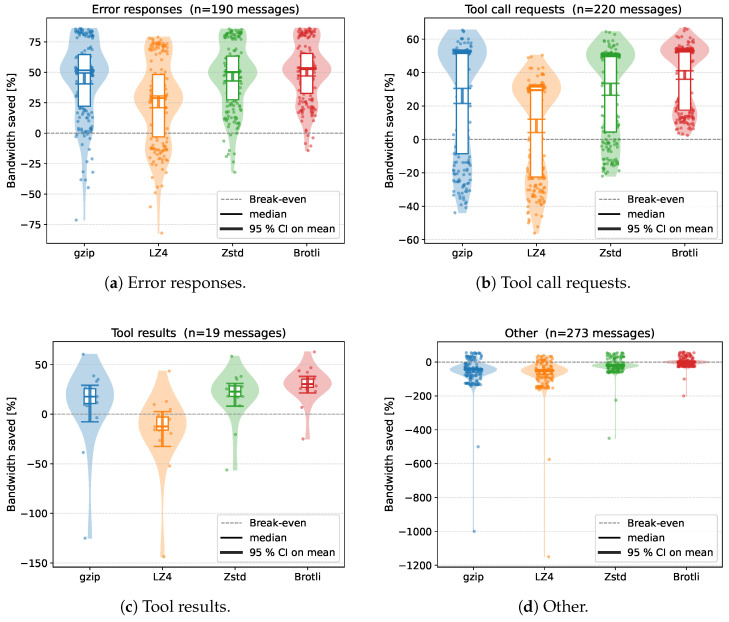
Compression savings by message type. Violin: Full distribution; dots: per-message medians; box: IQR; thick bar: 95% CI on the mean; dashed line: break-even (0%).

When evaluating the compression performance by message type in Figure 4, we initially note that all codecs compress larger messages quite effectively. Error responses are highly compressed by all codecs, with median savings of ≈53% for Brotli, ≈52% for gzip, ≈50% for Zstd, and ≈29% for LZ4. The 95% confidence interval (CI) for the mean sits above zero for all codecs. Tool-call requests follow the same ordering and are likewise net positive for every codec (median savings of ≈53%, ≈52%, ≈50%, and ≈30% for Brotli, gzip, Zstd, and LZ4, respectively).

The only category in which a codec illustrated in Figure 4 is net negative is “tool results,” where LZ4 has a median of ≈−12% while the other codecs stay positive. However, there are only 19 messages in this group, which results in wide CIs. The remaining messages fall in the “other” category (*n* = 273), which is dominated by short payloads below the per-codec break-even. Here, the individual message savings are negative for gzip, Zstd, and LZ4 and near zero for Brotli. The per-type differences therefore track each type’s underlying size distribution rather than an intrinsic codec property. This overall reinforces the size-driven characteristics we illustrated in Figure 2 and Figure 3.

### 3.3. MCP Atlas Dataset on Raspberry Pi4

For the Atlas dataset described in Section 2.1, we consider only the initial query and the final results response for low-powered and low-bandwidth connected devices, such as the RPi4 setup we employ and describe in Section 2.2, as an example. We initially provide an overview of attained compressions for queries and responses in Table 2.

We note that the general order of approaches remains similar to the prior observed ones, with LZ4 offering the least compression, and the remaining codecs performing better, with Brotli leading the compression. For our corpus, we note that close to half of the combined data can be saved through compression with either Zstd or Brotli, and about a quarter with LZ4.

Next, we consider the compression overhead regarding CPU utilization and closely correlated energy requirements. This represents the query compression with the RPi4 WiFi off. Table 3 provides the per-operation CPU overhead and incremental energy for n=100 runs. The net energy for an individual operation is derived as

$$E_{\mathrm{op}}=\frac{E_{\mathrm{net}}}{\left(500\;\mathrm{payloads}\cdot 50\;\mathrm{repeats}\right)}\cdot 1000,$$

where $E_{\mathrm{net}}=E_{\mathrm{gross}}-P_{\mathrm{idle}}\cdot t_{\mathrm{window}}$.

We note that the incremental CPU power draw is commonly high (approximately 500–525 mW) for all codecs, as the BCM2711 CPU of the RPi4 runs at essentially constant full-load power regardless of the algorithm to fix the measurement environment. In turn, the energy differences are purely duration-driven. We note that Brotli compression “costs” about 12 times more energy than LZ4 per operation. We also note that gzip decompression (13.3 μJ/op, 29.2 μs/op) is about twice as slow and twice as energy-intensive as Brotli decompression (7.2 μJ/op, 13.8 μs/op). This is an important asymmetry that should be considered for server-side receive paths. Overall, Zstd is the most energy-efficient codec with meaningful compression, with 17.4 μJ/op for 46.7% in bandwidth reduction.

Taking into account the energy variation between transmitting and receiving the compressed message in our testbed, we observe that the total energy range remains within the confidence interval, thereby confirming that the employed wireless setup is, overall, codec-invariant.

We next shift the view to the overall message latency, which in real-world deployments would be augmented by the remote inference. We derive the results presented in Table 4 by combining the compression of the query (CQ), decompression of the response (DR), and measured round-trip-time (RTT) to the end-to-end (E2E) metric and compare it to the uncompressed (raw) scenario.

On the employed 802.11ac WiFi, compression reduces E2E latency for most codecs. LZ4 and Zstd achieve 4–5% reductions as smaller compressed payloads reduce 802.11 queuing delay by more than the CPU overhead adds. In contrast, gzip has a significantly longer CPU time, which exactly offsets its RTT benefit, yielding neutral E2E.

### 3.4. MCP Atlas Dataset on Raspberry Pi Pico 2W

We repeated the Raspberry Pi4 setup by replacing it as DUT with a Raspberry Pi Pico 2W. As the MicroPython runtime due to memory limitations provides neither Zstd nor Brotli, we limit our evaluation to the Raw, gzip using the MicroPython deflate module (with a 512 B window), and LZ4 (Level 0, compiled as a native C module). We evaluate a 25-message subset of the 500 queries and 25 of the 500 responses, each repeated 20 times per run (vs. 50 on the RPi4) as a 16 kB payload cap is imposed by the 520 kB SRAM available on-device. We note that this subset is not size-stratified, so its absolute byte counts and compression savings are specific to the subset, however, the observed characteristics for energy and the wireless timing results for the 802.11n radio module are independent of corpus composition. We present the results in Table 5 for four batches of ten (we excluded a cold-start batch and used a separate radio-off run to determine the idle baseline).

As observed for the RPi4, the incremental power during compression and decompression is essentially constant across codecs: every codec draws 12–15 mW above the 62 mW idle baseline (radio-off). In turn, the per-operation energy is governed by processing time and the duration-driven energy model generalizes from the RPi4 application processor to the microcontroller in the Pico 2W. We note that while idle baseline and incremental power are roughly 36× lower in absolute terms, the MicroPython codecs are two to three orders of magnitude slower than the RPi4’s native implementations (LZ4 0.72 ms vs. 9.7 µs; gzip 52.7 ms vs. 77.8 µs per query), so the resulting energy cost of a compression operation is higher (LZ4 8.5 µJ/op and gzip 788 µJ/op, vs. 5.1 and 37.8 µJ/op on the RPi4).

In contrast to observations for the RPi4, we note that compression for the Pi Pico 2W saves transmission energy using the 802.11n radio module. As the 802.11n link is far slower than the RPi4’s 802.11ac (per-message RTT is about 21–41 ms vs. ≈3.6 ms), transmission energy scales with payload size. The radio’s incremental active power, denoted as ΔP in Table 6, is defined as the mean power drawn over the round-trip window minus the idle baseline ($\Delta P={\bar{P}}_{\mathrm{window}}-{\bar{P}}_{\mathrm{idle}}$). It is itself codec-invariant (≈116–120 mW). As shown in Table 6, the round-trip radio energy falls monotonically with the compressed byte count, up to over 40% reduction considering gzip vs. Raw. In contrast to the observations for the RPi4’s 802.11ac radio which can be approximated with a fixed radio cost, 802.11n is payload-size dependent and compression becomes energy-positive. We note that the absolute round-trip energies and the radio’s active power results in Table 6 are session-dependent. The table reports them as indicative, while the within-session 95% CIs stay ≤ 3%.

Shifting our view to the end-to-end latency by combining the previous results, we derive the equivalent to the RPi4 Table 4 for the Raspberry Pi Pico 2W in Table 7.

We note that LZ4 provides the lowest end-to-end latency (35.8 ms), beating both Raw (37.5 ms) and gzip (81.0 ms). On the Pico 2W microcontroller, the latency penalty of a heavy compression codec is severe, as gzip’s increased compression time is significantly more than the resulting RTT advantage. In turn, gzip is the slowest option end-to-end despite sending the fewest bytes.

Overall, the additional measurements for the Raspberry Pi Pico 2W confirm a duration-driven CPU energy model and show that the WiFi energy conclusion is link-specific. This enables compression for a slower, byte-proportional 802.11n link to recover transmission energy that 802.11ac hides for the evaluated scenario.

## 4. Discussion

Overall, we notice that the message size is the primary predictor of compression benefit. We observe savings near zero or negative below 100–200 B (depending on codec), and savings asymptotically approach 80–90% above 10 kB. We also note that LZ4 should not be used for typical MCP traffic without discrimination. With the highest break-even of the four evaluated codecs around 200 B, LZ4 expands rather than compresses the short tool call requests and results that dominate our analyzed corpus. We report these as illustration for the underlying size effects, noting that different use cases might yield different distributions of message sizes. Brotli achieves the highest compression ratio but also has the highest CPU cost. Zstd offers the best practical trade-off as it produces consistently positive savings from about 70 B upward and low CPU overhead. Overall, we can derive current general recommendations presented in Table 8.

While message classification might yield general tendencies for message size, the simplest approach remains to select based on available compute and energy at the end device as well as size. Overall, energy requirements for the wireless network (which we did not evaluate here) could erase gains for compression. Future research can evaluate the individual benefits for different processors and sensors in greater detail and derive composite schemes composed of different codec pairings based on use cases.

### 4.1. A Decision Rule for Practitioners

**Figure 5:**
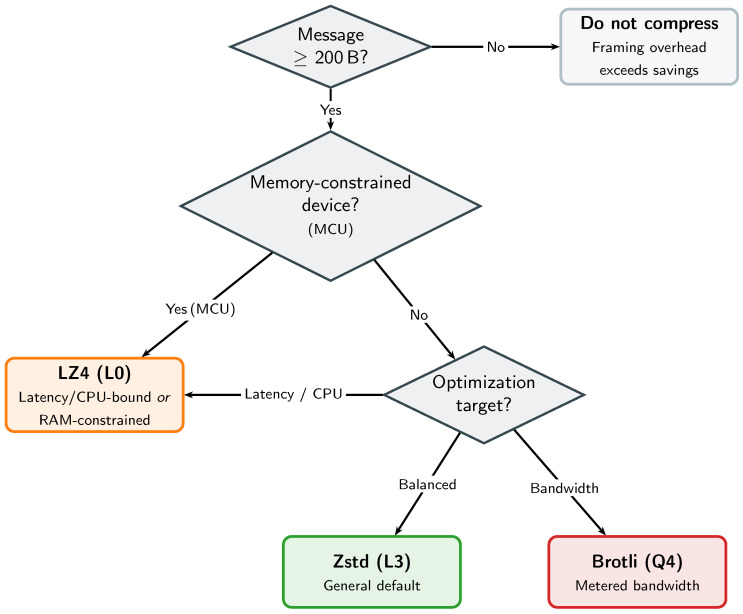
Codec-selection decision rule for MCP compression across device classes. Below the per-codec break-even (≈200 B) compression is skipped. On memory-constrained MCUs LZ4 is the only feasible codec, while otherwise the choice follows the optimization target.

Our measurements support a simple, threshold-based selection procedure for deploying compression in MCP systems. The governing variable is message size, and the secondary variable is the deployment’s optimization target (latency, bandwidth, or energy). In general, one should refrain from compression below a codec’s break-even size; we derive that a single conservative cutoff of 200 B avoids negative savings for every codec evaluated for our corpus. Zstd (Level 3) can be used as the general-purpose default, delivering above 40% bandwidth reduction at the best energy-per-byte of the codecs tested. If CPU or latency is the binding constraint, LZ4 (Level 0) should be used as it achieves a comparable end-to-end latency reduction. Brotli (Quality 4) should only be used when bandwidth is the scarce or metered resource as it incurs the greatest CPU energy. gzip only offers ubiquity as a merit. We summarize these quick guidelines in Figure 5.

### 4.2. Energy Recovery on Wireless

One key question is whether the compression bandwidth reduction yields net energy savings once wireless transmission is included. We find that for 802.11ac, incremental transmission energy is statistically identical across codecs and unrelated to payload size. Since transmission energy does not fall with a change in the compressed payload, the CPU cost of compression is never recovered, and every codec increases the total per-message energy vs. uncompressed. In turn, one could generalize that for high-speed wireless links, compression is only justified by latency and bandwidth, not energy. For lower-rate or byte-proportional wireless links (LoRa, NB-IoT, power-controlled cellular, saturated channels), our 802.11n result is the first empirical evidence that this could change significantly and is subject to our ongoing research.

### 4.3. MCP Implementation Considerations

MCP currently does not negotiate content-encoding and compression must be arranged out of band or below JSON-RPC. When MCP executes over HTTP(S), HTTP Content-Encoding and Accept-Encoding (using gzip, Brotli, and Zstd) require no changes and avoid double-compression. A preferred policy could be straightforward threshold-based, applying per-message compression independently above a size break-even point, capturing most benefits for large messages without penalizing small MCP messages. Per-session dictionaries might help with small repetitive messages, but add state and recovery complexity that we will evaluate in future work.

### 4.4. Cross-Scenario Synthesis and Limitations

Our approach separates codec benefit from cost and shows that cost is platform-dependent in ways benefit is not. On a more performant platform, we find a duration-driven CPU energy cost paired with wireless transmission energy that is payload-invariant. In turn, compression is justified by latency and bandwidth alone. The resource-constrained device is characterized by CPU usage paired with a transmission that is energy byte-proportional. In turn, the selection guidance bifurcates by platform and the embedded memory wall, which gates codec selection. The embedded codec options are memory-bounded and the application-class trade-off curves cannot be directly applied.

#### 4.4.1. Hardware Scope and Generalization Limits

Our primary measurements use a single application-class ARM platform (Raspberry Pi 4, BCM2711, Cortex-A72) and a smaller subset was evaluated for the Raspberry Pi Pico 2W (RP2350). Compression cost depends on architecture, ISA extensions, cache hierarchies, and power management, we subsequently note that absolute energy and latency results do not directly transfer to other architectures and configurations. However, some generalization can be noted in the relative order of algorithms through reliance on general compute time.

#### 4.4.2. Representativeness of the Message Corpora

We evaluate two specific corpora with controlled size characteristics. While we consider these to be representative of MCP traffic, we do not consider them to be exhaustive and representative for any general deployment. Crucially, the break-even thresholds and ratios we report are expressed as a function of message size, which is corpus-independent and therefore robust.

#### 4.4.3. Wireless Transmissions

The energy trade-off we report is specific to 802.11ac WiFi, on which transmission energy is dominated by the radio’s active-state power rather than payload size. On byte-proportional links such as the evaluated 802.11n, the balance differs and may favor compression. A further crossover toward LoRa/NB-IoT remains as part of future research.

## Figures and Tables

**Table 1:** Comparison of compression codecs employed.

Codec	Algorithm	Parameters	Role
Raw	No compression	—	Baseline
LZ4	LZ4 frame	Level 0 (fast)	Speed-optimized
Zstd	Zstandard	Level 3 (default)	Balanced
gzip	DEFLATE	Level 6	Compatibility
Brotli	Brotli	Quality 4	Ratio-optimized

**Table 2:** Compressions for queries, responses, and combined bandwidth savings attained for codecs used with the Atlas dataset.

Codec	Query Ratio	Response Ratio	Combined Savings
Raw	1.000	1.000	0.0%
LZ4	0.708	0.769	27.1%
Zstd	0.498	0.599	46.7%
gzip	0.487	0.603	47.3%
Brotli	0.468	0.563	49.9%

**Table 3:** Net operational overhead in time and energy in μJ for queries and responses with 95% confidence intervals.

Codec	Query (Compress)		Response (Decompress)	
	Time [μs/op]	Ener. [μJ/op]	Time [μs/op]	Ener. [μJ/op]
Raw	0.2	≈0	0.2	≈0
LZ4	9.7 ± <0.1	5.1 ± <0.1	3.5 ± <0.1	1.8 ± <0.1
Zstd	35.6 ± 0.2	17.4 ± <0.1	10.5 ± 0.1	4.8 ± <0.1
gzip	77.8 ± 0.3	37.8 ± <0.1	29.2 ± 0.1	13.3 ± <0.1
Brotli	125.1 ± 0.5	63.1 ± 0.1	13.8 ± 0.1	7.2 ± <0.1

**Table 4:** End-to-end per-message latency (*n* = 100).

Codec	CQ [μs]	RTT [ms]	DR [μs]	E2E [ms]	±CI	vs. Raw
Raw	0.2	3.791	0.2	3.792	0.121	—
LZ4	9.7	3.606	3.5	3.619	0.072	−4.6%
Zstd	35.6	3.566	10.5	3.612	0.207	−4.7%
gzip	77.8	3.703	29.2	3.810	0.413	+0.5%
Brotli	125.1	3.619	13.8	3.758	0.234	−0.9%

**Table 5:** CPU values for the Raspberry Pi Pico 2W with 95% CIs (n=40 in four batches).

Codec	Query (Compress)		Response (Decompress)	
	Time [ms/op]	Ener. [μJ/op]	Time [ms/op]	Ener. [μJ/op]
Raw	0.009	≈0	0.007	≈0
LZ4	0.72	8.5 ± 0.4	0.47	5.4 ± 0.4
gzip	52.7	788 ± 14	7.2	110 ± 3

**Table 6:** Round-trip metrics on the Raspberry Pi Pico 2W (n=40), bytes are per direction. Absolute round-trip energy and radio ΔP (mean incremental power over the round-trip window relative to the idle baseline) are RF-session-dependent (shown as ≈ as they varied across sessions); the byte counts, the byte-proportional ordering, and the within-session 95% CIs (≤ 3%) are stable.

Codec	Bytes	RTT Mean [ms]	Round-Trip Energy [mJ]	Δ*P* [mW]
Raw	36,436	37.4	≈110	≈117
LZ4	24,358	34.6	≈100	≈116
gzip	21,761	21.1	≈64	≈120

**Table 7:** End-to-end per-message latency for the Raspberry Pi Pico 2W (*n* = 40).

Codec	CQ [ms]	RTT [ms]	DR [ms]	E2E [ms]	vs. Raw
Raw	0.009	≈37.4	0.007	≈37.5	—
LZ4	0.72	≈34.6	0.47	≈35.8	−4.5%
gzip	52.7	≈21.1	7.2	≈81.0	+116%

**Table 8:** Codec trade-off summary for the RPi4.

Codec	Savings	E2E	Compress	Decompress	Use Case
Raw	0 %	3.79 ms	≈0	≈0	Very small payloads
LZ4	27 %	3.62 ms	5.1 μJ	1.8 μJ	Latency-critical
Zstd	47 %	3.61 ms	17.4 μJ	4.8 μJ	General-purpose
gzip	47 %	3.81 ms	37.8 μJ	13.3 μJ	Interoperability only
Brotli	50 %	3.76 ms	63.1 μJ	7.2 μJ	Bandwidth-constrained

## Data Availability

The MCP trace dataset analyzed in this study is publicly available at https://huggingface.co/datasets/IvanHU/MCP-Tools_calls-Trace (accessed on 5 July 2026) [14]. The MCP-Atlas benchmark dataset is publicly available at https://huggingface.co/datasets/ScaleAI/MCP-Atlas (accessed on 5 July 2026) [16]. The energy and timing measurements generated in this study are available from https://doi.org/10.17605/OSF.IO/YTEVQ.

## Abbreviations

The following abbreviations are used in this manuscript:

API	Application Programming Interface
CPU	Central Processing Unit
DUT	Device Under Test
E2E	End-to-End
HTML	Hypertext Markup Language
HTTP	HyperText Transfer Protocol
IETF	Internet Engineering Task Force
JSON	JavaScript Object Notation
LLM	Large Language Model
MCP	Model Context Protocol
OAS	OpenAPI Specifications
REST	Representational State Transfer
RFC	Request For Comments
RPC	Remote Procedure Call
RPi4	Raspberry Pi 4
RTT	Round-Trip-Time
Zstd	Zstandard
